# Eye movement characteristics in a mental rotation task presented in virtual reality

**DOI:** 10.3389/fnins.2023.1143006

**Published:** 2023-03-27

**Authors:** Zhili Tang, Xiaoyu Liu, Hongqiang Huo, Min Tang, Xiaofeng Qiao, Duo Chen, Ying Dong, Linyuan Fan, Jinghui Wang, Xin Du, Jieyi Guo, Shan Tian, Yubo Fan

**Affiliations:** ^1^Key Laboratory for Biomechanics and Mechanobiology of Ministry of Education, Beijing Advanced Innovation Center for Biomedical Engineering, School of Biological Science and Medical Engineering and School of Engineering Medicine, Beihang University, Beijing, China; ^2^State Key Laboratory of Virtual Reality Technology and Systems, Beihang University, Beijing, China

**Keywords:** eye movements, virtual reality, naturalistic stimuli, mental rotation, three-dimensional stimuli, visual perception

## Abstract

**Introduction:**

Eye-tracking technology provides a reliable and cost-effective approach to characterize mental representation according to specific patterns. Mental rotation tasks, referring to the mental representation and transformation of visual information, have been widely used to examine visuospatial ability. In these tasks, participants visually perceive three-dimensional (3D) objects and mentally rotate them until they identify whether the paired objects are identical or mirrored. In most studies, 3D objects are presented using two-dimensional (2D) images on a computer screen. Currently, visual neuroscience tends to investigate visual behavior responding to naturalistic stimuli rather than image stimuli. Virtual reality (VR) is an emerging technology used to provide naturalistic stimuli, allowing the investigation of behavioral features in an immersive environment similar to the real world. However, mental rotation tasks using 3D objects in immersive VR have been rarely reported.

**Methods:**

Here, we designed a VR mental rotation task using 3D stimuli presented in a head-mounted display (HMD). An eye tracker incorporated into the HMD was used to examine eye movement characteristics during the task synchronically. The stimuli were virtual paired objects oriented at specific angular disparities (0, 60, 120, and 180°). We recruited thirty-three participants who were required to determine whether the paired 3D objects were identical or mirrored.

**Results:**

Behavioral results demonstrated that the response times when comparing mirrored objects were longer than identical objects. Eye-movement results showed that the percent fixation time, the number of within-object fixations, and the number of saccades for the mirrored objects were significantly lower than that for the identical objects, providing further explanations for the behavioral results.

**Discussion:**

In the present work, we examined behavioral and eye movement characteristics during a VR mental rotation task using 3D stimuli. Significant differences were observed in response times and eye movement metrics between identical and mirrored objects. The eye movement data provided further explanation for the behavioral results in the VR mental rotation task.

## 1. Introduction

Mental rotation is the ability to mentally represent and rotate two-dimensional (2D) images or three-dimensional (3D) objects (Shepard and Metzler, 1971) and has been widely used to examine visuospatial ability (Pletzer et al., 2019; Ito et al., 2022). In classical mental rotation tasks, participants visually perceive 3D objects and mentally rotate them until the objects are identified. It is generally accepted that the mental rotation process includes five cognitive stages (Desrocher et al., 1995): (i) processing of visual information and creating mental images of the presented objects (imagining and evaluating the presented objects from different angles); (ii) mentally rotating the objects or images; (iii) comparing the presented objects; (iv) determining whether the presented objects are identical; (v) making a decision (indicated by a button). Response times and accuracy rates are widely employed in examining a mental rotation effect and mental rotation performance (Shepard and Metzler, 1971; Berneiser et al., 2018), but these behavioral indices are not sufficient to fully understand the related complex cognitive processes. Recent studies have provided evidence that eye movement characteristics are promising for examining these mental processes (Xue et al., 2017; Toth and Campbell, 2019; Tiwari et al., 2021).

Eye-tracking technology is an effective tool for examining cognitive processes required for complex cognitive tasks (Lancry-Dayan et al., 2023). Eye movements captured by eye tracking systems can provide comprehensive information on mental processes in mental rotation tasks and have been effective in revealing brain activity (Toth and Campbell, 2019). It has been suggested that eye movement parameters could characterize mental representation according to specific patterns (Nazareth et al., 2019). Eye movement metrics, including fixations and saccades, have been used in studies using mental rotation tasks (Suzuki et al., 2018). Identifying fixations allows researchers to examine objects of interest (Mast and Kosslyn, 2002); fixations are mainly responsible for the acquisition of visual information in mental rotation tasks (Yarbus, 1967). A recent study indicated that fixation metrics could illustrate mental rotation strategies, and that fixation patterns were related to mental rotation performance (Nazareth et al., 2019). Saccades are the rapid eye movements between fixations (Kowler et al., 1995). Saccades serve to rapidly shift the fovea to a new target to integrate visual information from fixations. The integration allows a brain to compare the visual information obtained from fixations with the remembered image of the object (Ibbotson and Krekelberg, 2011). In addition, eye tracking data can be used to characterize different mental rotation strategies. Holistic and piecemeal strategies have been extensively investigated in previous studies on mental rotation (Khooshabeh et al., 2013; Hsing et al., 2023). The holistic strategy refers to mentally rotating one of two 3D objects as a whole and encoding the spatial information of the object. For instance, when comparing two objects, one object is holistically rotated along a vertical axis for comparison with the other object. The piecemeal strategy refers to segmenting an object into several pieces and encoding only part of its spatial information. For instance, one of the two objects would be segmented into several independent pieces, and participants may mentally rotate one piece of both objects and see if the two pieces match. Strategy ratio was commonly used to reflect which strategies are performed during mental rotation (Khooshabeh and Hegarty, 2010). The strategy ratio refers to the ratio of the number of fixations within an object to the number of saccades between the two objects. In a holistic strategy, the ratio would be 1; in a piecemeal strategy, the ratio would be greater than one. Although previous studies have provided insights into eye movement characteristics in mental rotation tasks, these findings have mainly used 2D images presented on computer screens (Xue et al., 2017; Campbell et al., 2018). Previously, the use of visual stimuli has relied heavily on simplified image stimuli (Snow and Culham, 2021), which are distinctly different from naturalistic stimuli.

Recently, visual neuroscience studies have focused on visual behavior in response to naturalistic stimuli rather than simplified images (Haxby et al., 2020; Jaaskelainen et al., 2021; Musz et al., 2022). Visual behavior and brain activity evoked by planar images are different from those evoked by natural 3D objects (Marini et al., 2019; Chiquet et al., 2020). Natural 3D objects are rich in depth cues and visual input from the surrounding environment (e.g., edges) (Chiquet et al., 2020). For example, a recent study showed that 3D objects triggered more stronger brain responses than 2D images do (Marini et al., 2019; Tang et al., 2022).

Virtual Reality (VR) is an emerging technology used to provide naturalistic stimuli, allowing us to understand behavioral characteristics in an immersive environment similar to real world (Hofmann et al., 2021). This technology serves to fill the gap between traditional presentations based on 2D computer screen and naturalistic visual presentations close to real world (Wenk et al., 2022). Virtual environments can simulate real-world visual inputs (Robertson et al., 1993; Minderer and Harvey, 2016), allowing more naturalistic 3D objects with depth cues (El Jamiy and Marsh, 2019). Comparing with the mental rotation tasks using 2D images, the VR version of mental rotation task using 3D objects provides new opportunities to quantify visuospatial ability in environments similar to the real world. For example, a recent work has shown the impact of virtual environments on mental rotation performance. They assessed the differences in mental rotation ability based on dimensionality and the complexity of virtual environments, which provided new insights into the impact of stereo 3D objects on mental rotation performance (Lochhead et al., 2022). Their results suggested that the performance advantage of 3D objects could be greater than that of conventional 2D mediums. In addition, the previous work of our lab demonstrated the behavioral performance and neural oscillations in the mental rotation task using 2D images and using stereoscopic 3D objects (Tang et al., 2022). These studies inspired some expanded studies related to the VR version of mental rotation task, such as exploration of eye movement characteristics. Eye-tracking technology was incorporated into a head-mounted display (HMD), allowing to provide a new opportunity to understand human visual behavior in VR (Clay et al., 2019; Chiquet et al., 2020). Eye movement characteristics based on 3D objects help to understand visual behavior in a natural context close to real world (Chiquet et al., 2020). However, the eye movement characteristics in mental rotation tasks using naturalistic 3D objects with depth cues have been rarely reported.

Here, we designed a VR mental rotation task using 3D stimuli presented in a HMD with an eye tracker to examine the eye movements during the task synchronically. We first used behavioral performance to validate a mental rotation effect presented in a VR environment. Further, we highlighted the eye movement characteristics in this task. As we expected, our results indicated that the VR task also evoke a mental rotation effect, suggesting that the virtual task was effective and available. We analyzed the eye movement data to explain the behavioral results observed in the VR mental rotation task.

## 2. Materials and methods

### 2.1. Participants

Thirty-three participants were enrolled [17 females and 16 males; mean age = 28.59 years, standard deviation (SD) = 2.41]. All participants were recruited from university, were right-handed, and had normal or corrected-to normal vision. Written informed consent was obtained from all participants, and ethical approval was granted by the local ethical committee in accordance with the Declaration of Helsinki. Data from one participant was excluded because of low eye tracking data quality. Thus, data from 32 participants were included in the analysis.

### 2.2. Construction of 3D visual stimuli

**FIGURE 1:**
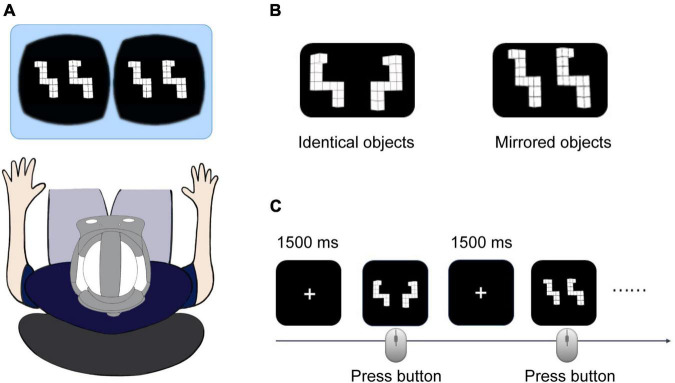
**(A)** Rendered 3D visual stimuli presented by a head-mounted display (HMD). **(B)** Sample of three-dimensional (3D) identical and mirrored stimuli. **(C)** Schematic diagram of the mental rotation task. The participants were asked to judge whether the paired objects were identical or mirrored and respond by pressing a mouse button (left: identical and right: mirrored) as quickly and accurately as possible.

We constructed 120 pairs of 3D objects based on original stimuli from the mental-rotation stimulus library (the 4th prototype) (Peters and Battista, 2008). The 3D paired objects were oriented at specific angular disparities (0, 60, 120, and 180^°^). For each pair, the two stimuli were identical or mirrored objects (Figure 1B). The 120 stimuli consisted of pairs of objects (identical, mirrored) in four angular disparities (0, 60, 120, and 180^°^).

Each 3D object was constructed using 3D Studio Max (Autodesk Inc., San Rafael, CA, USA) and stored as .obj files. We used the Unity game engine (Unity Software, Inc., San Francisco, CA, USA) to render the 3D objects. The rendered 3D visual stimuli were presented in a HMD (VIVE Pro Eye; HTC Corporation, Taipei, Taiwan) in randomized sequences [generated using MATLAB (The MathWorks, Natick, MA, USA)].

### 2.3. Experiment procedure

All participants completed the VR mental rotation task with 3D stimuli presented in the HMD in a quiet room (Figure 1A). Participants’ information, including demographic data and VR experience, were collected before the experiment. During the experiment, participants were seated comfortably in a chair. The experimenter put a VR headset on the participants’ head, and a five-point calibration of the eye tracker was performed before the experiment. We started the experiment when the calibrations were successful.

The VR mental rotation task consisted of 120 trials separated into two blocks of 60 trials. Short breaks of approximate 5 min were assigned between blocks to prevent participant fatigue. For each trial, a white fixation cross was displayed in the center of the HMD for 1,500 ms, followed by the presentation of a pair of 3D visual stimuli. The participants were asked to judge whether the paired objects were identical or mirrored and to respond by pressing a mouse button (left button for identical and right button for mirrored) as quickly and accurately as possible. The trial ended once the participants indicated their decision by pressing a button (Figure 1C).

### 2.4. Behavioral data acquisition and analysis

We used a customized script in the Unity 3D platform to record behavioral data including response times and accuracy rates. Stimulus onsets and trial completion times were marked by the script. The response time was defined as the duration from stimulus onset to trial completion. The accuracy rate was defined as the ratio of trials correctly judged out of the total number of trials.

### 2.5. Eye movement data acquisition and analysis

The eye tracker was incorporated into the HMD. Eye movement data were collected using an eye tracking SDK (SRanipal), with a maximum frequency of 120 Hz. A collider (i.e., a Unity object) was added to the surface of each 3D object presented in the HMD. The eye tracker in the HMD could capture all the possible gazes on the surface of the collider.

Eye movement events, including fixations and saccades, were identified to represent the eye movement characteristics in the VR mental rotation task. For each trial, we applied an identification by dispersion threshold (IDT) algorithm to group the collected gaze data into fixations (Salvucci and Goldberg, 2000; Llanes-Jurado et al., 2020). The end of each fixation was marked as a saccade event. The IDT algorithm requires two parameters: the dispersion threshold and the minimum fixation duration (Blignaut, 2009). This algorithm has been found to be the most similar to manual detection from human experts (Andersson et al., 2017). In the present study, fixations were marked using a 1^°^ spatial dispersion threshold (Blignaut, 2009; Arthur et al., 2021) and a minimum duration of 60 ms (Komogortsev et al., 2010). To test the reliability of the results presented in our study, we used the eye movement metrics to compare the eye movement results obtained from the IDT algorithm with the three dispersion thresholds (1, 1.2, and 1.4^°^). Further analyses showed that the eye movement results obtained from the dispersion threshold of 1^°^ were consistent with that from the dispersion thresholds of 1.2 and 1.4^°^, respectively (Supplementary Figures 1–4). Detailed statistical results are listed in Supplementary Tables 1–4. These results justify the dispersion threshold of 1^°^ in our study.

**FIGURE 2:**
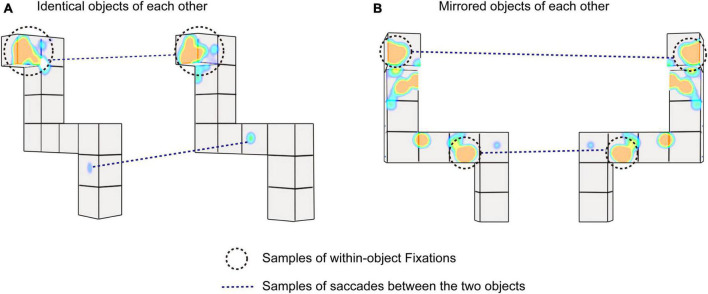
Examples of eye movements during the virtual reality (VR) mental rotation. **(A)** Sample three-dimensional (3D) identical stimuli. **(B)** Sample 3D mirrored stimuli. Black circles with dotted lines indicate samples of fixations within one object. Dotted lines indicate samples of saccades between the two objects.

Four eye movement metrics were used based on prior studies. The percent fixation time, the number of within-object fixations, the number of saccades, and the strategy ratio were used to quantify visual behavior (Khooshabeh and Hegarty, 2010; Khooshabeh et al., 2013; Lavoie et al., 2018; Nazareth et al., 2019; Hsing et al., 2023). Examples of the eye movement metrics (e.g., fixations and saccades) are presented in Figure 2. The eye movement metrics were defined as follows.

#### 2.5.1. Percent fixation time

The amount of time fixated on the two objects during the mental rotation task divided by the total duration of the task (i.e., response time), multiplied by 100.

#### 2.5.2. Number of within-object fixations

The number of fixations per second made on either of the two objects.

#### 2.5.3. Number of saccades

The number of saccades that a participant made from one 3D object to another within 1 s.

#### 2.5.4. Strategy ratio

The ratio of the number of fixations within an object to the number of saccades made between the two objects. The strategy ratio was used to reflect holistic or piecemeal strategies. A strategy ratio close to one indicates a holistic strategy, and a strategy ratio greater than one suggests a piecemeal strategy.

### 2.6. Statistical analysis

All statistical analyses were conducted using SPSS software (IBM Corp., Armonk, NY, USA). Two-way repeated-measures analysis of variance (ANOVA) was used to analyze behavioral metrics (response time and accuracy rate) and eye movement metrics (fixations and saccades). Stimulus Type (identical and mirrored) and Angular Disparity (0, 60, 120, and 180^°^) served as within-subject factors. Sphericity was examined using Mauchly’s test of sphericity; if the assumption of sphericity had been violated, Greenhouse-Geisser correction were reported. Bonferroni correction was used to account for multiple comparisons. The effect size was evaluated using partial eta squared ($\eta_{p}^{2}$). All data are presented as mean ± standard error of the mean (SE). The detailed statistical results about ANOVA were listed in Supplementary Table 5.

## 3. Results

### 3.1. Behavioral results

#### 3.1.1. Response time

**FIGURE 3:**
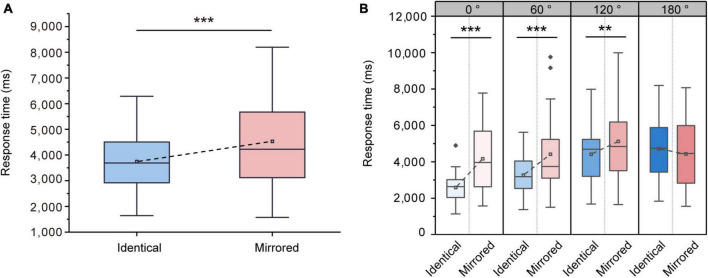
Changes in response times during the virtual reality (VR) mental rotation task. **(A)** Boxplots present response times for identical and mirrored objects. **(B)** Grouped boxplots present response times for identical and mirrored objects at 0, 60, 120 and 180^°^. The boxplots illustrate the first quartile, median, and third quartile and 1.5 times the interquartile range for both the upper and lower ends of the box. Black horizontal lines and asterisks denote significant differences (**p < 0.01, ***p < 0.001).

Response times were used to examine whether there is a mental rotation effect during the VR task. The ANOVA on the response times showed significant main effects of Stimulus Type [F (1, 31) = 22.147, *p* < 0.001, $\eta_{p}^{2}$ = 0.417] and Angular Disparity [F (3, 93) = 39.530, *p* < 0.001, $\eta_{p}^{2}$ = 0.560]. The significant main effect of Stimulus Type was caused by lower response times when presented with identical objects (3746.63 ± 207.94 ms, mean ± SE) compared to mirrored objects (4532.23 ± 327.65 ms, mean ± SE; Figure 3A). The interaction between Stimulus Type and Angular Disparity was also significant [F (3, 93) = 17.854, *p* < 0.001, $\eta_{p}^{2}$ = 0.365]. Pairwise comparisons revealed significant differences in response times between identical and mirrored objects at 0, 60 and 120^°^ (all *p* < 0.01; Table 1 and Figure 3B). There was no significant difference in response times between identical and mirrored objects at 180° (*p* = 0.077; Table 1 and Figure 3B).

**TABLE 1:** Overview of response times (mean ± standard error) for identical and mirrored objects at four angular disparities.

	Identical (ms)	Mirrored (ms)	Statistics
0^°^	2586.57 ± 139.26	4166.23 ± 328.10	F (1, 31) = 40.307, *p* < 0.001, $\eta_{p}^{2}$ = 0.565
60^°^	3268.35 ± 186.32	4415.54 ± 363.68	F (1, 31) = 18.313, *p* < 0.001, $\eta_{p}^{2}$ = 0.371
120^°^	4416.90 ± 269.35	5122.03 ± 378.53	F (1, 31) = 8.235, *p <* 0.01, $\eta_{p}^{2}$ = 0.210
180^°^	4714.69 ± 313.53	4425.12 ± 333.83	F (1, 31) = 3.345, *p* = 0.077, $\eta_{p}^{2}$ = 0.097

#### 3.1.2. Accuracy rate

**FIGURE 4:**
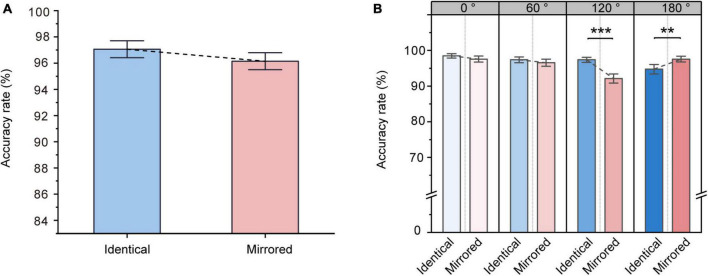
Changes in accuracy rates during the virtual reality (VR) mental rotation task. **(A)** Bar graphs present average accuracy rates for identical and mirrored objects. **(B)** Grouped bar graphs present accuracy rates for identical and mirrored objects at 0, 60, 120 and 180^°^. Error bars denote standard errors. Black lines and asterisks denote significant differences (**p < 0.01, ***p < 0.001).

The ANOVA on the accuracy rates showed no significant main effect of Stimulus Type [F (1, 31) = 2.519, *p* = 0.123, $\eta_{p}^{2}$ = 0.075]. The accuracy rates for identical objects (97.05 ± 0.64 %, mean ± SE) were not significantly higher than that for mirrored objects (96.14 ± 0.65 %, mean ± SE; Figure 4A). There was a significant main effect of Angular Disparity [F (3, 93) = 5.255, *p* = 0.002, $\eta_{p}^{2}$ = 0.145] and a significant interaction between Angular Disparity and Stimulus Type [F (3, 93) = 11.873, *p* < 0.001, $\eta_{p}^{2}$ = 0.277]. The differences in different angular disparity between identical and mirrored objects were further revealed. Pairwise comparisons revealed significant differences in accuracy rates between identical and mirrored objects at 120 and 180^°^ (all *p* < 0.01; Table 2 and Figure 4B). There was no difference (all *p* > 0.05; Table 2 and Figure 4B) in accuracy rates between identical and mirrored objects at 0 and 60^°^.

**TABLE 2:** Accuracy rates (mean ± standard error) for identical and mirrored objects at four angular disparities.

	Identical (%)	Mirrored (%)	Statistics
0^°^	98.43 ± 0.61	98.13 ± 0.68	F (1, 31) = 0.129, *p* = 0.721, $\eta_{p}^{2}$ = 0.004
60^°^	97.29 ± 0.84	96.46 ± 1.04	F (1, 31) = 0.392, *p* = 0.536, $\eta_{p}^{2}$ = 0.012
120^°^	97.71 ± 0.64	92.08 ± 1.32	F (1, 31) = 29.160, *p <* 0.001, $\eta_{p}^{2}$ = 0.485
180^°^	94.79 ± 1.36	97.91 ± 0.75	F (1, 31) = 9.992, *p <* 0.01, $\eta_{p}^{2}$ = 0.242

### 3.2. Eye movement results

#### 3.2.1. Percent fixation time

**FIGURE 5:**
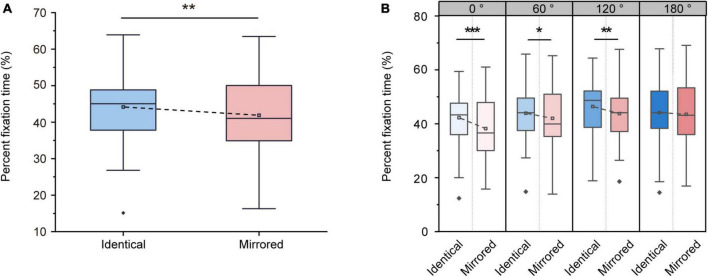
Changes in percent fixation time during the virtual reality (VR) mental rotation task. **(A)** Boxplots present percent fixation times for identical and mirrored objects. **(B)** Grouped boxplots present percent fixation times for identical and mirrored objects at 0, 60, 120 and 180^°^. The boxplots illustrate the first quartile, median, and third quartile and 1.5 times the interquartile range for both the upper and lower ends of the box. Black horizontal lines and asterisks denote significant differences (*p < 0.05, **p < 0.01, ***p < 0.001).

The ANOVA on the percent fixation time revealed significant main effects of Stimulus Type [F (1, 31) = 11.658, *p* = 0.002, $\eta_{p}^{2}$ = 0.273] and Angular Disparity [F (3, 93) = 14.334, *p* < 0.001, $\eta_{p}^{2}$ = 0.316]. The significant main effect of Stimulus Type demonstrated that the percent fixation time was significantly higher for identical objects (44.18 ± 1.84 %, mean ± SE) than for mirrored objects (41.87 ± 2.11 %, mean ± SE; Figure 5A). Moreover, there was also a significant interaction between Angular Disparity and Stimulus Type [F (3, 93) = 2.789, *p* = 0.045, $\eta_{p}^{2}$ = 0.083]. Pairwise comparisons revealed significant differences between identical and mirrored objects at 0, 60 and 120^°^ (all *p* < 0.05; Table 3 and Figure 5B). There was no difference in the percent fixation time (*p* = 0.531; Table 3 and Figure 5B) between identical and mirrored objects at 180^°^.

**TABLE 3:** Overview of percent fixation time (mean ± standard error) for identical and mirrored objects at four angular disparities.

	Identical (%)	Mirrored (%)	Statistics
0^°^	42.24 ± 1.87	38.20 ± 2.24	F (1, 31) = 11.919, *p* < 0.01, $\eta_{p}^{2}$ = 0.278
60^°^	43.90 ± 1.97	41.99 ± 2.19	F (1, 31) = 4.558, *p* < 0.05, $\eta_{p}^{2}$ = 0.128
120^°^	46.39 ± 1.86	43.78 ± 1.99	F (1, 31) = 9.134, *p <* 0.01, $\eta_{p}^{2}$ = 0.228
180^°^	44.16 ± 2.07	43.51 ± 2.28	F (1, 31) = 0.402, *p* = 0.531, $\eta_{p}^{2}$ = 0.013

#### 3.2.2. Number of within-object fixations

**FIGURE 6:**
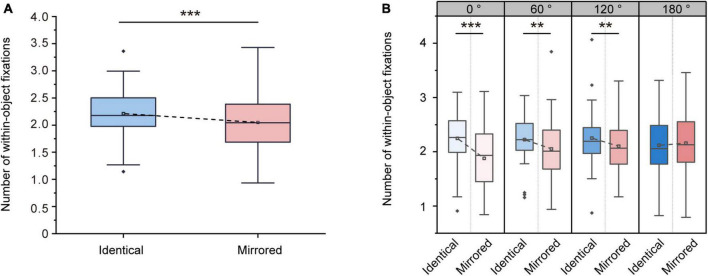
Changes in number of within-object fixations during the virtual reality (VR) mental rotation task. **(A)** Boxplots present the number of within-object fixations for identical and mirrored objects. **(B)** Grouped boxplots present the number of within-object fixations for identical and mirrored objects at 0, 60, 120 and 180^°^. The boxplots illustrate the first quartile, median, and third quartile and 1.5 times the interquartile range for both the upper and lower ends of the box. Black horizontal lines and asterisks denote significant differences (**p < 0.01, ***p < 0.001).

Repeated-measures ANOVA on the number of within-object fixations revealed a significant main effect of Stimulus Type [F (1, 31) = 32.853, *p* < 0.001, $\eta_{p}^{2}$ = 0.515]. The number of within-object fixations was significantly higher for identical objects (2.21 ± 0.08, mean ± SE) than for mirrored objects (2.05 ± 0.09, mean ± SE; Figure 6A), suggesting that the participants might make multiple comparisons within an object when comparing identical objects. This was akin to a piecemeal strategy (Khooshabeh et al., 2013), in which the participants might break the objects into pieces and encode partial spatial information. We also observed a significant main effect of Angular Disparity [F (3, 93) = 3.225, *p* = 0.026, $\eta_{p}^{2}$ = 0.094] and a significant interaction between Angular Disparity and Stimulus Type [F (3, 93) = 11.929, *p* < 0.001, $\eta_{p}^{2}$ = 0.278]. Pairwise comparisons revealed significant differences in the number of within-object fixations between identical and mirrored objects at 0, 60 and 120^°^ (all *p* < 0.01; Table 4 and Figure 6B). However, there was no difference (*p* = 0.394; Table 4 and Figure 6B) in the number of within-object fixations between identical and mirrored objects at 180^°^.

**TABLE 4:** Overview of number of within-object fixations (mean ± standard error) for identical and mirrored objects at four angular disparities.

	Identical	Mirrored	Statistics
0^°^	2.25 ± 0.08	1.88 ± 0.10	F (1, 31) = 37.476, *p* < 0.001, $\eta_{p}^{2}$ = 0.547
60^°^	2.22 ± 0.07	2.05 ± 0.10	F (1, 31) = 12.646, *p* < 0.01, $\eta_{p}^{2}$ = 0.290
120^°^	2.25 ± 0.09	2.10 ± 0.09	F (1, 31) = 9.907, *p <* 0.01, $\eta_{p}^{2}$ = 0.242
180^°^	2.12 ± 0.09	2.15 ± 0.10	F (1, 31) = 0.746, *p* = 0.394, $\eta_{p}^{2}$ = 0.024

#### 3.2.3. Number of saccades

**FIGURE 7:**
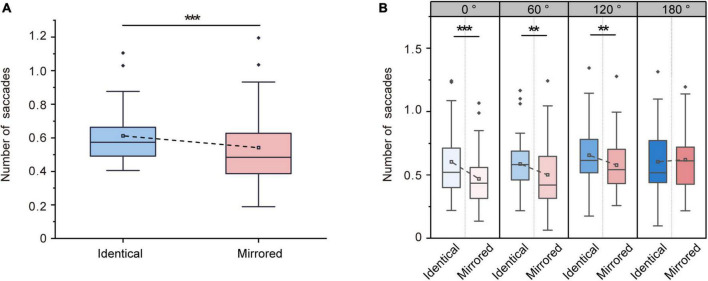
Changes in number of saccades during the virtual reality (VR) mental rotation task. **(A)** Boxplots present the number of saccades for identical and mirrored objects. **(B)** Grouped boxplots present the number of saccades for identical and mirrored objects at 0, 60, 120 and 180^°^. The boxplots illustrate the first quartile, median, and third quartile and 1.5 times the interquartile range for both the upper and lower ends of the box. Black horizontal lines and asterisks denote significant differences (**p < 0.01, ***p < 0.001).

Repeated-measures ANOVA revealed significant main effects of Stimulus Type [F (1, 31) = 20.299, *p* < 0.001, $\eta_{p}^{2}$ = 0.396] and Angular Disparity [F (3, 93) = 11.343, *p* < 0.001, $\eta_{p}^{2}$ = 0.268]. The significant main effect of Stimulus Type was due to a higher number of saccades for identical objects (0.61 ± 0.03, mean ± SE) compared to for mirrored objects (0.54 ± 0.04, mean ± SE; Figure 7A). A significant interaction between Angular Disparity and Stimulus Type was also observed [F (3, 93) = 6.760, *p* < 0.001, $\eta_{p}^{2}$ = 0.179]. Pairwise comparisons revealed significant differences in the number of saccades between identical and mirrored objects at 0, 60 and 120^°^ (all *p* < 0.01; Table 5 and Figure 7B). However, there was no difference in the number of saccades (*p* = 0.595; Table 5 and Figure 7B) between identical and mirrored objects at 180^°^.

**TABLE 5:** Overview of number of saccades (mean ± standard error) for identical and mirrored stimuli at four angular disparities.

	Identical	Mirrored	Statistics
0^°^	0.60 ± 0.04	0.46 ± 0.04	F (1, 31) = 25.221, *p* < 0.001, $\eta_{p}^{2}$ = 0.449
60^°^	0.58 ± 0.04	0.50 ± 0.04	F (1, 31) = 10.733, *p* < 0.01, $\eta_{p}^{2}$ = 0.257
120^°^	0.65 ± 0.04	0.57 ± 0.03	F (1, 31) = 12.330, *p <* 0.01, $\eta_{p}^{2}$ = 0.285
180^°^	0.60 ± 0.04	0.62 ± 0.04	F (1, 31) = 0.288, *p* = 0.595, $\eta_{p}^{2}$ = 0.009

#### 3.2.4. Strategy ratio

**FIGURE 8:**
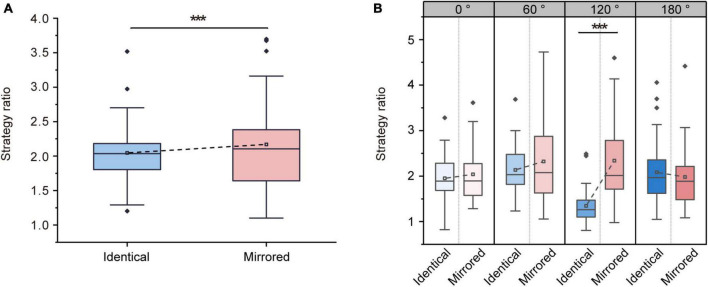
Changes in strategy ratio during the virtual reality (VR) mental rotation task. **(A)** Boxplots present average strategy ratio for identical and mirrored objects. **(B)** Grouped boxplots present strategy ratio for identical and mirrored objects at 0, 60, 120 and 180^°^. The boxplots illustrate the first quartile, median, and third quartile and 1.5 times the interquartile range for both the upper and lower ends of the box. Black horizontal lines and asterisks denote significant differences (***p < 0.001).

Repeated-measures ANOVA on strategy ratios revealed significant main effects of Stimulus Type [F (1, 31) = 17.008, *p* < 0.001, $\eta_{p}^{2}$ = 0.354] and Angular Disparity [F (3, 93) = 9.987, *p* < 0.001, $\eta_{p}^{2}$ = 0.244]. The significant main effect of Stimulus Type demonstrated that the strategy ratio for identical objects (2.04 ± 0.08, mean ± SE) was significantly lower than that for mirrored objects (2.17 ± 0.12, mean ± SE; Figure 8A). Moreover, there was a significant interaction between Angular Disparity and Stimulus Type [F (3, 93) = 23.035, *p* < 0.001, $\eta_{p}^{2}$ = 0.426]. Pairwise comparisons revealed significant differences in strategy ratios between identical and mirrored objects at 120° (*p* < 0.001; Table 6 and Figure 8B). There was no difference in strategy ratios (*p* > 0.05; Table 6 and Figure 8B) between identical and mirrored objects at 0, 60 and 180^°^.

**TABLE 6:** Overview of strategy ratio (mean ± standard error) for identical and mirrored objects at four angular disparities.

	Identical	Mirrored	Statistics
0^°^	1.95 ± 0.09	2.03 ± 0.11	F (1, 31) = 0.643, *p* = 0.429, $\eta_{p}^{2}$ = 0.020
60^°^	2.13 ± 0.09	2.32 ± 0.14	F (1, 31) = 2.083, *p* = 0.159, $\eta_{p}^{2}$ = 0.063
120^°^	1.34 ± 0.06	2.34 ± 0.15	F (1, 31) = 70.104, *p <* 0.001, $\eta_{p}^{2}$ = 0.693
180^°^	2.08 ± 0.13	1.97 ± 0.12	F (1, 31) = 1.145, *p* = 0.243, $\eta_{p}^{2}$ = 0.044

## 4. Discussion

In the present study, we presented behavioral and eye movement characteristics during a VR mental rotation task using 3D objects. We found that the VR mental rotation task also evoked a mental rotation effect. Significant differences were observed in response times and eye movement metrics between identical and mirrored objects. The eye movement data further explained the reasons for that response times were longer when comparing mirrored objects than when comparing identical objects.

Based on mental rotation task using 2D images, we conducted a VR mental rotation task using 3D objects. The 2D images presented *via* a computer screen provide an illusion of depth (Snow and Culham, 2021). Compared to 2D images of the 3D objects (Griksiene et al., 2019; Toth and Campbell, 2019), 3D objects presented in VR can provide real depth cues, enhancing the realness of visual stimuli (Tang et al., 2022). VR allows balances between experimental control and ecological validity (Snow and Culham, 2021). The stereo 3D objects presented in VR in the current task are more ecologically valid than simple 2D images, which can improve our understanding of the neural mechanisms associated with naturalistic visual stimuli. Previously, some studies have compared the metal rotation performance of 2D and stereo 3D forms (Neubauer et al., 2010; Price and Lee, 2010; Lochhead et al., 2022; Tang et al., 2022). Although some factors (i.e., experimental paradigm, sample size, and experimental environment) may affect the experimental results of comparisons between conventional 2D and stereo 3D forms, these studies may provide support for the VR mental rotation task with eye tracking.

As expected, we observed a significant mental rotation effect (Shepard and Metzler, 1971), indicating that the VR mental rotation task is effective and available. Interestingly, the identical and mirrored stimuli are different in behavioral performance, which was consistent with literatures (Hamm et al., 2004; Paschke et al., 2012; Chen et al., 2014). Our results demonstrated that the accuracy rate for identical objects decreased at 180° and the accuracy rate for mirrored objects increased at 180°, which were line with the findings of a prior study (Chen et al., 2014). The differences in accuracy rate between identical and mirrored objects might be associated with the differences in cognitive processing and a decrease of the task difficulty at higher angular disparities for mirrored objects (Paschke et al., 2012). Due to opposite arm positions, the paired mirrored stimulus at 180^°^ could be directly perceived as mirrored object without any rotation manipulation. That is, the participants probably determined the mirrored objects by only comparing the arm positions of objects, which might increase correct responses. By contrast, rotation operation is required at 120^°^. Response-preparation theory (Cooper and Shepard, 1973) suggested that motor response during mental rotation was planned on the basis of the expectancy of the paired objects being identical. For mirrored objects, the expectancy may result in that an already planned motor response would have to be inhibited and re-planned. Moreover, the expectancy would contribute to a lower accuracy rate because the planned motor responses were probably difficult to inhabit. Therefore, the accuracy rates for identical objects are higher than that for mirrored objects at 120^°^. Response times were higher when comparing mirrored objects than when comparing identical objects, which might be attributed to additional cognitive processing. In the 3D mental rotation task, participants were asked to decide whether the paired objects were identical or mirrored. The participants mentally represented the paired objects and rotated them and simultaneously made a direct match between their internal mental representation and the external visual stimuli. A decision of “mirrored” would be made if the participants discovered that some parts of the paired objects were different. When the participants identified differences between objects, more response time was required to choose the “mirrored” response. This was possibly associated with the strategy of visual processing during mental rotation (Toth and Campbell, 2019), which was further supported by the eye tracking data.

Eye movements allow us to scan the visual field with high resolution (Mast and Kosslyn, 2002), and can provide insights into visual behavior in mental rotation task using 2D images (Nazareth et al., 2019). However, few studies have reported eye movements in mental rotation tasks involving naturalistic 3D stimuli. In the present work, we analyzed eye movement parameters, including fixations and saccades, to quantify the processes of visual processing of 3D objects. We found that percent fixation time was higher when participants compared identical objects than mirrored objects. Eye fixations provide information on active processing of information (Mast and Kosslyn, 2002) and are associated with visual attention (Verghese et al., 2019; Skaramagkas et al., 2023). The 3D objects were encoded at each fixation and was then reconstructed in the brain based on input from multiple fixations. Effective visual information can be extracted around these fixations (Ikeda and Takeuchi, 1975). The higher percent fixation times for identical objects suggested that the participants spent more time fixating on the objects, indicating that the participants might allocate more attention to the objects during the completion of the mental rotation task when comparing the identical objects. The increased attention suggested that more visual information was obtained when the participants compared identical 3D objects. In addition, the number of fixations made on 3D objects per second was higher for identical objects than for mirrored objects, suggesting that the participants might make more fixations per second when comparing identical objects. Because visual information was obtained from each fixation, the participants dealt with more visual information per second when comparing the identical objects. This may indicate that the efficiency of visual information acquisition was higher for identical objects than for mirrored objects. These results further illustrated that faster response times for identical objects could be attributed to increased attention and higher efficiency of visual information acquisition.

Visual strategies during mental rotation have been associated with behavioral performance (Heil and Jansen-Osmann, 2008). Strategy dichotomies, including holistic and piecemeal strategies, have been extensively discussed in previous studies (Heil and Jansen-Osmann, 2008; Khooshabeh et al., 2013). The piecemeal strategy generally results in longer response times compared with the holistic strategy. These different visual strategies can be reflected by the strategy ratio, which is the ratio of the number of within-object fixations to the number of between-object saccades. The present study showed a difference in strategy ratios between mirrored objects and identical objects. Previous studies demonstrated that a strategy ratio close to 1 indicated a holistic strategy and a strategy ratio greater than one indicated a piecemeal strategy (Khooshabeh and Hegarty, 2010). Thus, the participants in the present study preferred a piecemeal strategy for mirrored objects compared with identical objects. For mirrored objects, participants performed multiple fixations within one object to compare different parts of the object before they switched to the other. Fixation switches were more frequently observed when comparing mirrored objects than when comparing identical objects. Fixation switches between the two 3D objects could be regard as constant updates, which is necessary to maintain the object perception (Hyun and Luck, 2007). These constant updates may be more difficult during the mental rotation of mirrored objects. These findings provided further explanation for the behavioral results observed in the VR mental rotation task.

This study presents the behavioral and eye movement characteristics in a VR mental rotation task with eye tracking, but it still has some limitations. Although the present VR mental rotation task elicits a mental rotation effect, comparing the characteristics of VR to 2D mental rotation task is also important. The comparisons could provide compelling evidence that whether the VR mental rotation task is a better alternative. Future studies could add a conventional 2D mental rotation task with eye tracking and compare the differences in behavioral and eye movement characteristics between 2D and VR mental rotation tasks.

## 5. Conclusion

In the present work, we examined behavioral and eye movement characteristics during a VR mental rotation task using 3D stimuli. Significant differences were obtained in behavioral performance and eye movement metrics in the rotation and comparison of identical and mirrored objects. Eye movement metrics, including the percent fixation time, the number of within-object fixations, and the number of saccades, were significantly lower when comparing mirrored objects than identical objects. The eye movement data provided further explanation for the behavioral results in the VR mental rotation task.

## Data availability statement

The original contributions presented in this study are included in the article/Supplementary material, further inquiries can be directed to the corresponding authors.

## Ethics statement

The studies involving human participants were reviewed and approved by the current study adhered to the tenets of the Declaration of Helsinki, and the ethical approval was approved by Beihang University (BM20200183). All of the participants provided written informed consents in advance of the study. A signed informed consent statement was received from each participant. The patients/participants provided their written informed consent to participate in this study.

## Author contributions

ZT: formal analysis, writing—original draft, and visualization. XL: conceptualization, funding acquisition, project administration, and writing—review and editing. HH and XD: formal analysis. MT and LF: software. XQ, DC, JW, and JG: visualization. YD: writing—original draft. ST: writing—review and editing. YF: conceptualization, funding acquisition, project administration, supervision, and writing—review and editing. All authors contributed to the article and approved the submitted version.
