# Supplementary material for: Eye movement characteristics in a mental rotation task presented in virtual reality
Source: Front Neurosci. 2023 Mar 27;17:1143006. doi: 10.3389/fnins.2023.1143006 (PMC10083294; doi:10.3389/fnins.2023.1143006)
Supplement: Supplementary file 1 [file Data_Sheet_1.PDF]

Supplementary Material

Eye movement characteristics in a mental rotation task presented in virtual reality

Zhili Tang, Xiaoyu Liu, Hongqiang Huo, Min Tang, Xiaofeng Qiao, Duo Chen, Ying Dong, Linyuan Fan, Jinghui Wang, Xin Du, Jieyi Guo, Shan Tian, Yubo Fan

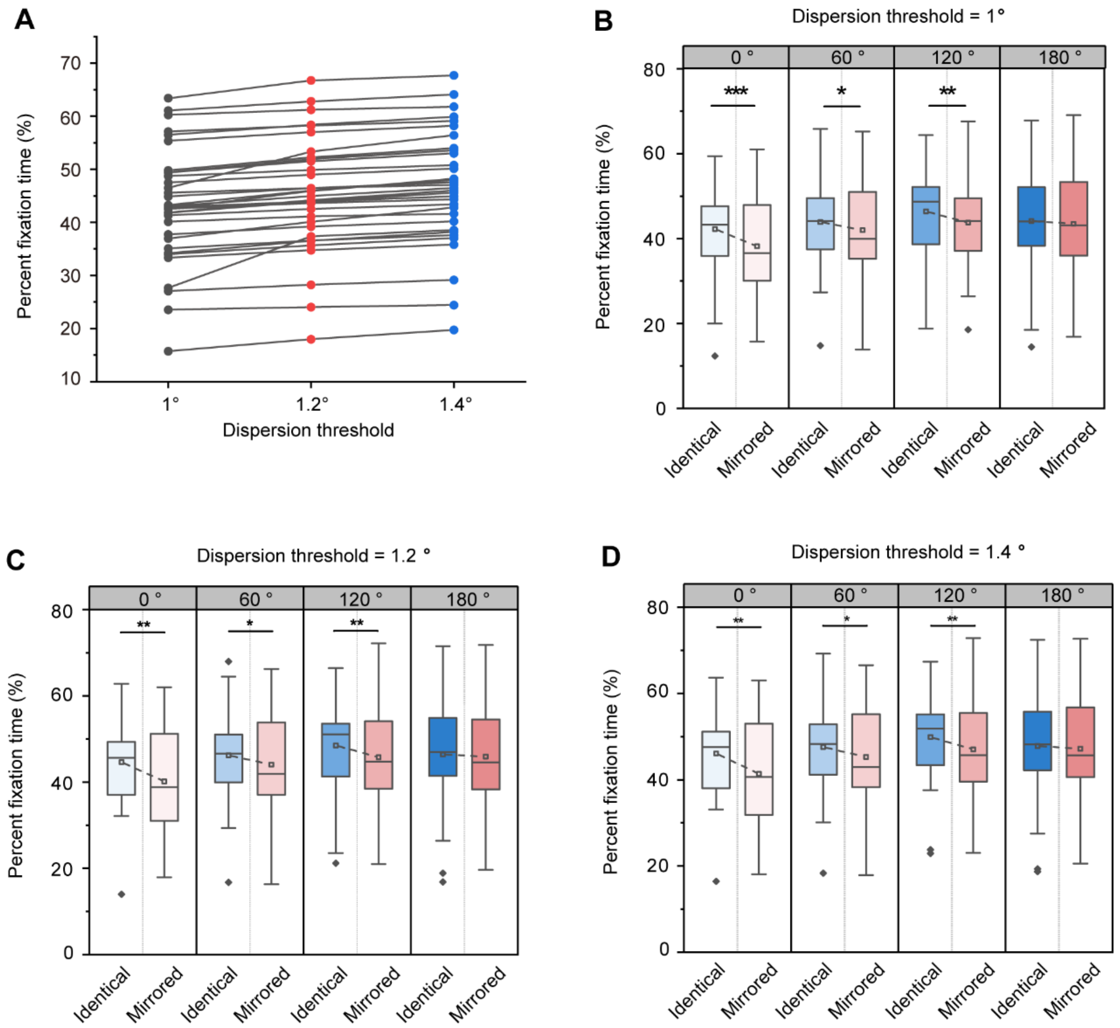

Supplementary Figure 1. Percent fixation time at three dispersion thresholds. (A) The percent fixation time at the dispersion thresholds of 1°, 1.2°, and 1.4°. Each dot represents the data of each participant from all trials at the dispersion thresholds of 1°, 1.2°, and 1.4° (Black: 1°; Red: 1.2°; Blue: 1.4°). Grouped boxplots present percent fixation time for identical and mirrored objects at 0°, 60°, 120° and 180° at the dispersion thresholds of 1° (B), 1.2° (C), and 1.4° (D). The boxplots illustrate the first quartile, median, and third quartile and 1.5 times the interquartile range for both the upper and lower ends of the box. Black horizontal lines and asterisks denote significant differences ($**p < 0.01$, $***p < 0.001$).

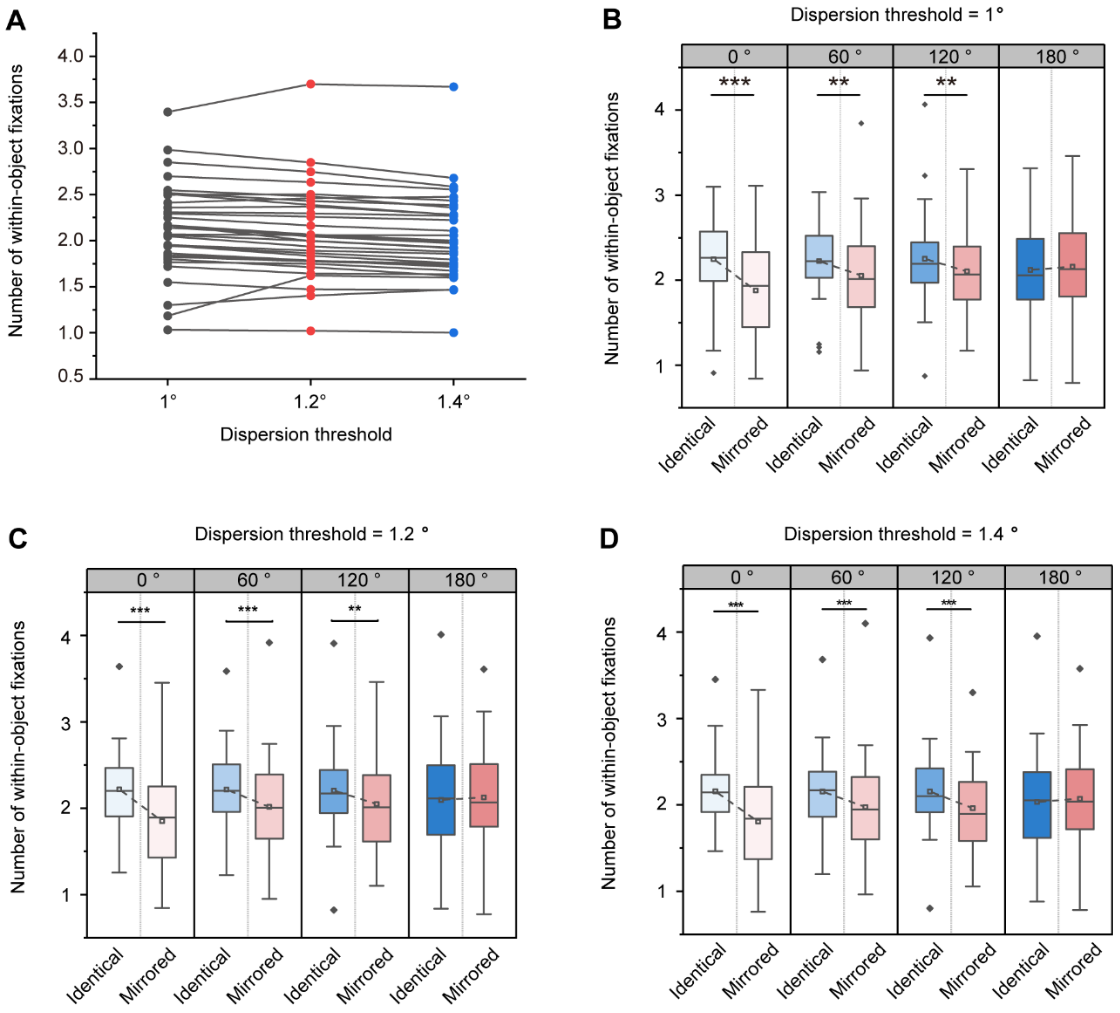

Supplementary Figure 2. Number of within-object fixations at three dispersion thresholds. (A) The number of within-object fixations at the dispersion thresholds of 1°, 1.2°, and 1.4°. Each dot represents the data of each participant from all trials at the dispersion thresholds of 1°, 1.2°, and 1.4° (Black: 1°; Red: 1.2°; Blue: 1.4°). Grouped boxplots present the number of within-object fixations for identical and mirrored objects at 0°, 60°, 120° and 180° at the dispersion thresholds of 1° (B), 1.2° (C), and 1.4° (D). The boxplots illustrate the first quartile, median, and third quartile and 1.5 times the interquartile range for both the upper and lower ends of the box. Black horizontal lines and asterisks denote significant differences (∗∗$p < 0.01$, ∗∗∗$p < 0.001$).

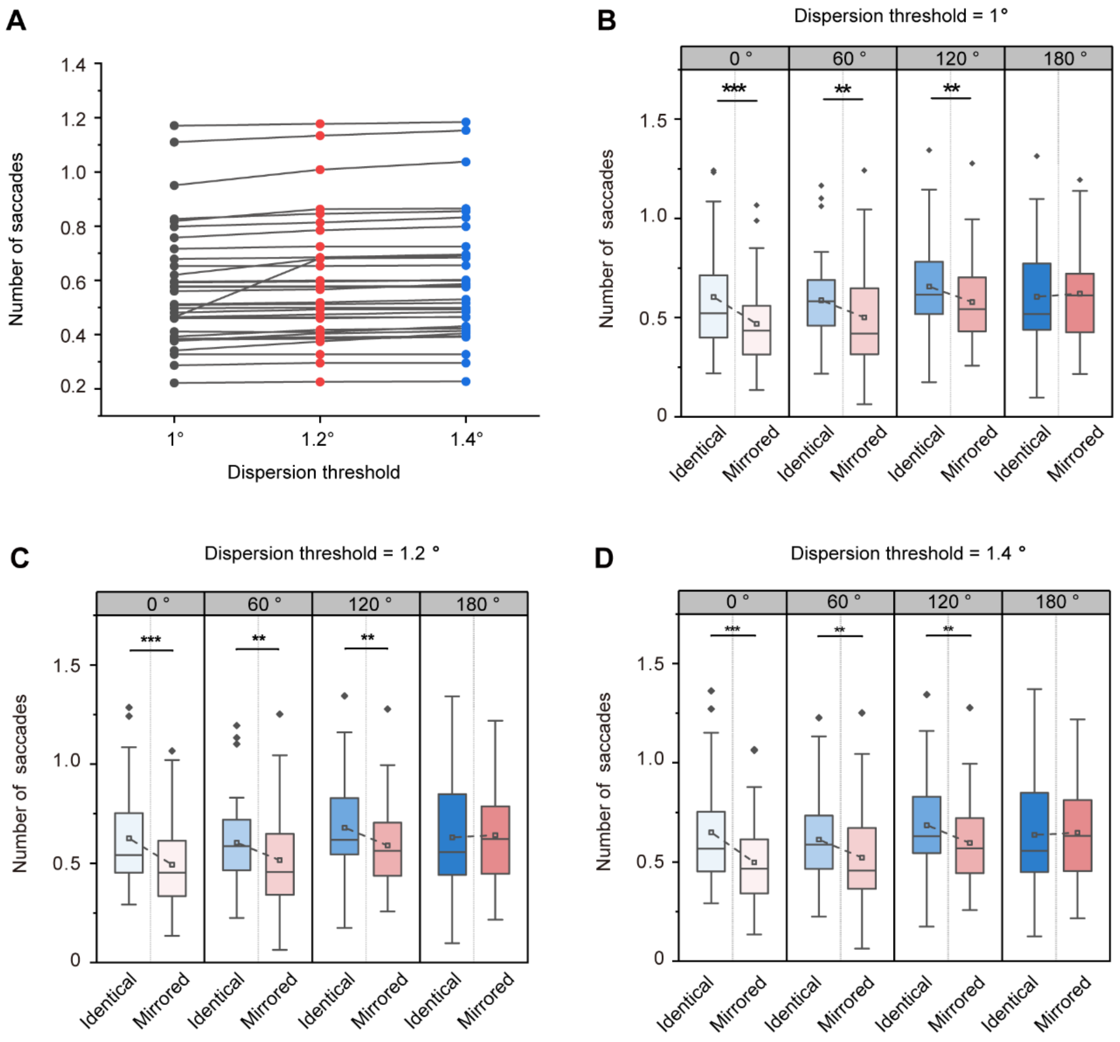

Supplementary Figure 3. Number of saccades at three dispersion thresholds. (A) The number of saccades at the dispersion thresholds of 1°, 1.2°, and 1.4°. Each dot represents the data of each participant from all trials at the dispersion thresholds of 1°, 1.2°, and 1.4° (Black: 1°; Red: 1.2°; Blue: 1.4°). Grouped boxplots present the number of saccades for identical and mirrored objects at 0°, 60°, 120° and 180° at the dispersion thresholds of 1° (B), 1.2° (C), and 1.4° (D). The boxplots illustrate the first quartile, median, and third quartile and 1.5 times the interquartile range for both the upper and lower ends of the box. Black horizontal lines and asterisks denote significant differences ($**p < 0.01$, $***p < 0.001$).

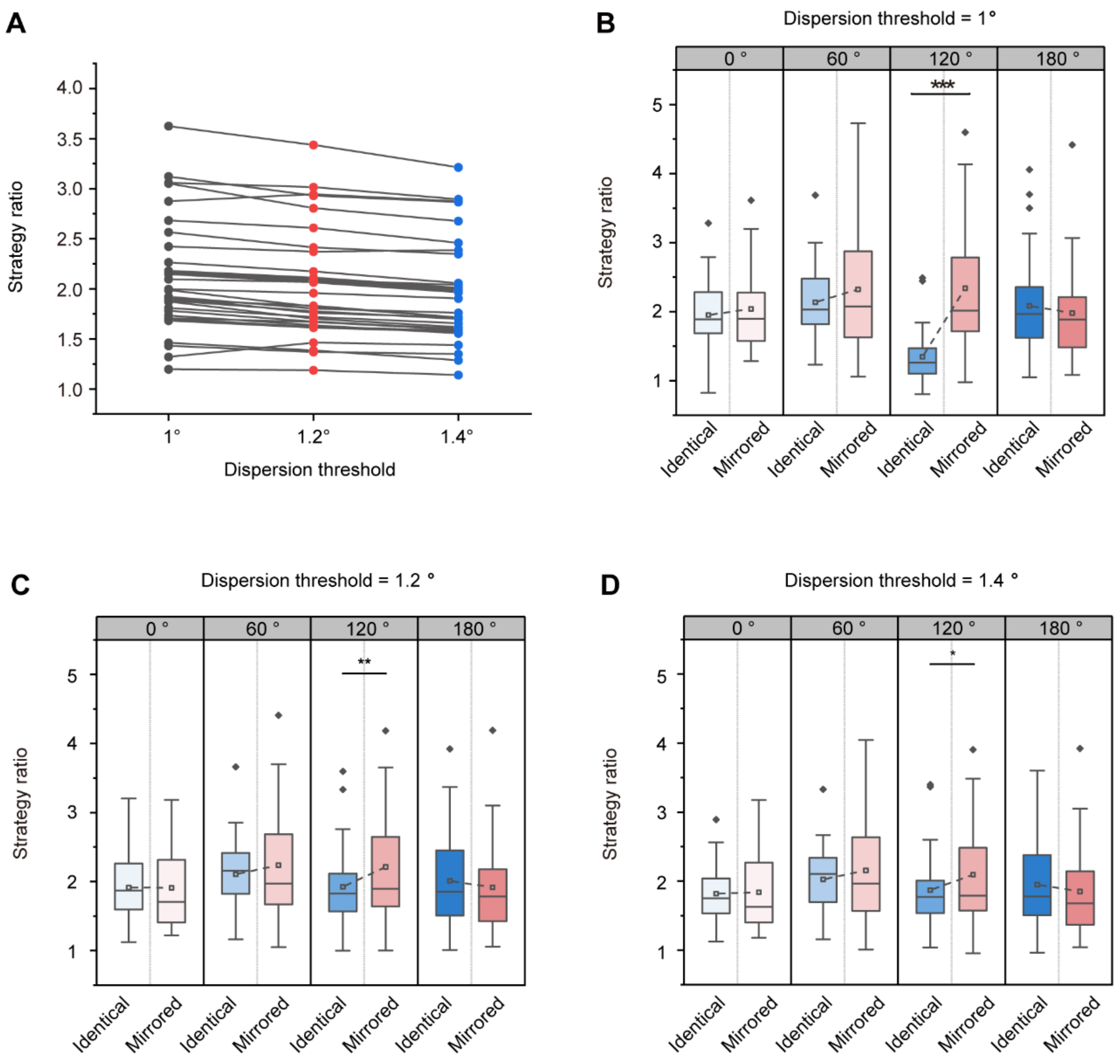

Supplementary Figure 4. Strategy ratio at three dispersion thresholds. (A) The strategy ratio at the dispersion thresholds of 1°, 1.2°, and 1.4°. Each dot represents the data of each participant from all trials at the dispersion thresholds of 1°, 1.2°, and 1.4° (Black: 1°; Red: 1.2°; Blue: 1.4°). Grouped boxplots present the strategy ratio for identical and mirrored objects at 0°, 60°, 120° and 180° at the dispersion thresholds of 1° (B), 1.2° (C), and 1.4° (D). The boxplots illustrate the first quartile, median, and third quartile and 1.5 times the interquartile range for both the upper and lower ends of the box. Black horizontal lines and asterisks denote significant differences ($**p < 0.01$, $***p < 0.001$).

Supplementary Table 1 Overview of the percent fixation time (mean ± standard error) for identical and mirrored objects in four angular disparity at the dispersion thresholds of 1°, 1.2°, and 1.4°

Dispersion threshold		Identical (%)	Mirrored (%)	Statistics
1°	0°	42.24 ± 1.87	38.20 ± 2.24	F (1, 31) = 11.919, $p < 0.01$, $\eta_p^2 = 0.278$
	60°	43.90 ± 1.97	41.99 ± 2.19	F (1, 31) = 4.558, $p < 0.05$, $\eta_p^2 = 0.128$
	120°	46.39 ± 1.86	43.78 ± 1.99	F (1, 31) = 9.134, $p < 0.01$, $\eta_p^2 = 0.228$
	180°	44.16 ± 2.07	43.51 ± 2.28	F (1, 31) = 0.402, $p = 0.531$, $\eta_p^2 = 0.013$
1.2°	0°	44.66 ± 1.77	40.16 ± 2.26	F (1, 31) = 14.165, $p < 0.01$, $\eta_p^2 = 0.314$
	60°	46.22 ± 1.92	44.05 ± 2.19	F (1, 31) = 4.908, $p < 0.05$, $\eta_p^2 = 0.137$
	120°	48.47 ± 1.86	45.77 ± 2.03	F (1, 31) = 9.181, $p < 0.01$, $\eta_p^2 = 0.228$
	180°	46.41 ± 2.09	45.89 ± 2.23	F (1, 31) = 0.276, $p = 0.603$, $\eta_p^2 = 0.009$
1.4°	0°	46.09 ± 1.77	41.36 ± 2.92	F (1, 31) = 15.594, $p < 0.001$, $\eta_p^2 = 0.335$
	60°	47.59 ± 1.93	45.28 ± 2.20	F (1, 31) = 5.631, $p < 0.05$, $\eta_p^2 = 0.154$
	120°	49.88 ± 1.88	47.03 ± 2.03	F (1, 31) = 10.095, $p < 0.01$, $\eta_p^2 = 0.246$
	180°	47.92 ± 2.01	47.18 ± 2.25	F (1, 31) = 0.366, $p = 0.550$, $\eta_p^2 = 0.012$

Supplementary Table 2 Overview of the number of within-object fixations (mean ± standard error) for identical and mirrored objects in four angular disparity at the dispersion thresholds of 1°, 1.2°, and 1.4°

Dispersion threshold		Identical (%)	Mirrored (%)	Statistics
1°	0°	2.25 ± 0.08	1.88 ± 0.10	F (1, 31) = 37.476, $p < 0.001$, $\eta_p^2 = 0.547$
	60°	2.22 ± 0.07	2.05 ± 0.10	F (1, 31) = 12.646, $p < 0.01$, $\eta_p^2 = 0.290$
	120°	2.25 ± 0.09	2.10 ± 0.09	F (1, 31) = 9.907, $p < 0.01$, $\eta_p^2 = 0.242$
	180°	2.12 ± 0.09	2.15 ± 0.10	F (1, 31) = 0.746, $p = 0.394$, $\eta_p^2 = 0.024$
1.2°	0°	2.21 ± 0.08	1.85 ± 0.10	F (1, 31) = 49.655, $p < 0.001$, $\eta_p^2 = 0.616$
	60°	2.21 ± 0.08	2.01 ± 0.10	F (1, 31) = 24.937, $p < 0.001$, $\eta_p^2 = 0.446$
	120°	2.20 ± 0.09	2.04 ± 0.08	F (1, 31) = 11.477, $p < 0.01$, $\eta_p^2 = 0.270$
	180°	2.09 ± 0.10	2.12 ± 0.09	F (1, 31) = 0.584, $p = 0.451$, $\eta_p^2 = 0.018$
1.4°	0°	2.15 ± 0.07	1.80 ± 0.09	F (1, 31) = 46.172, $p < 0.001$, $\eta_p^2 = 0.598$
	60°	2.15 ± 0.07	1.97 ± 0.10	F (1, 31) = 17.461, $p < 0.001$, $\eta_p^2 = 0.360$
	120°	2.15 ± 0.09	1.96 ± 0.08	F (1, 31) = 19.769, $p < 0.001$, $\eta_p^2 = 0.389$
	180°	2.03 ± 0.10	2.07 ± 0.09	F (1, 31) = 0.792, $p = 0.380$, $\eta_p^2 = 0.025$

Supplementary Table 3 Overview of the number of saccades (mean ± standard error) for identical and mirrored objects in four angular disparity at the dispersion thresholds of 1°, 1.2°, and 1.4°

Dispersion threshold		Identical (%)	Mirrored (%)	Statistics
1°	0°	0.60 ± 0.04	0.46 ± 0.04	$F(1, 31) = 25.221, p < 0.001, \eta_p^2 = 0.449$
	60°	0.58 ± 0.04	0.50 ± 0.04	$F(1, 31) = 10.733, p < 0.01, \eta_p^2 = 0.257$
	120°	0.65 ± 0.04	0.57 ± 0.03	$F(1, 31) = 12.330, p < 0.01, \eta_p^2 = 0.285$
	180°	0.60 ± 0.04	0.62 ± 0.04	$F(1, 31) = 0.288, p = 0.595, \eta_p^2 = 0.009$
1.2°	0°	0.62 ± 0.04	0.49 ± 0.04	$F(1, 31) = 30.772, p < 0.001, \eta_p^2 = 0.498$
	60°	0.60 ± 0.04	0.51 ± 0.04	$F(1, 31) = 11.708, p < 0.01, \eta_p^2 = 0.274$
	120°	0.67 ± 0.04	0.59 ± 0.04	$F(1, 31) = 13.770, p < 0.01, \eta_p^2 = 0.308$
	180°	0.63 ± 0.05	0.64 ± 0.04	$F(1, 31) = 0.130, p = 0.721, \eta_p^2 = 0.004$
1.4°	0°	0.64 ± 0.04	0.49 ± 0.04	$F(1, 31) = 36.425, p < 0.001, \eta_p^2 = 0.540$
	60°	0.61 ± 0.04	0.52 ± 0.04	$F(1, 31) = 12.784, p < 0.01, \eta_p^2 = 0.292$
	120°	0.68 ± 0.04	0.59 ± 0.04	$F(1, 31) = 14.024, p < 0.01, \eta_p^2 = 0.311$
	180°	0.63 ± 0.05	0.64 ± 0.04	$F(1, 31) = 0.122, p = 0.729, \eta_p^2 = 0.004$

Supplementary Table 4 Overview of the strategy ratio (mean ± standard error) for identical and mirrored objects in four angular disparity at the dispersion thresholds of 1°, 1.2°, and 1.4°

Dispersion threshold		Identical (%)	Mirrored (%)	Statistics
1°	0°	1.95 ± 0.09	2.03 ± 0.11	F (1, 31) = 0.643, p = 0.429, η_p^2 = 0.020
	60°	2.13 ± 0.09	2.32 ± 0.14	F (1, 31) = 2.083, p = 0.159, η_p^2 = 0.063
	120°	1.34 ± 0.06	2.34 ± 0.15	F (1, 31) = 70.104, p < 0.001, η_p^2 = 0.693
	180°	2.08 ± 0.13	1.97 ± 0.12	F (1, 31) = 1.145, p = 0.243, η_p^2 = 0.044
1.2°	0°	1.91 ± 0.08	1.91 ± 0.10	F (1, 31) = 0.001, p = 0.972, η_p^2 = 0.000
	60°	2.10 ± 0.09	2.23 ± 0.13	F (1, 31) = 1.259, p = 0.270, η_p^2 = 0.039
	120°	1.92 ± 0.09	2.21 ± 0.14	F (1, 31) = 9.225, p < 0.01, η_p^2 = 0.229
	180°	2.01 ± 0.12	1.91 ± 0.12	F (1, 31) = 1.244, p = 0.273, η_p^2 = 0.039
1.4°	0°	1.82 ± 0.07	1.83 ± 0.10	F (1, 31) = 0.039, p = 0.845, η_p^2 = 0.001
	60°	2.02 ± 0.08	2.15 ± 0.13	F (1, 31) = 1.351, p = 0.254, η_p^2 = 0.042
	120°	1.86 ± 0.09	2.02 ± 0.13	F (1, 31) = 5.873, p < 0.05, η_p^2 = 0.159
	180°	1.94 ± 0.12	1.85 ± 0.11	F (1, 31) = 1.570, p = 0.220, η_p^2 = 0.048

Supplementary Table 5 Summary of ANOVA results on behavioral and eye-movement data

	df	*F*	*p*	η_p^2
Behavioral data				
Response time				
Stimulus Type (T)	(1, 31)	22.147	< 0.001	0.417
Angular Disparity (A)	(3, 93)	39.530	< 0.001	0.560
T × A	(3, 93)	17.854	< 0.001	0.365
Accuracy rate				
Stimulus Type (T)	(1, 31)	2.519	0.123	0.075
Angular Disparity (A)	(3, 93)	5.255	0.002	0.145
T × A	(3, 93)	11.873	< 0.001	0.277
Percent fixation time				
Stimulus Type (T)	(1, 31)	11.658	0.002	0.273
Angular Disparity (A)	(3, 93)	14.334	< 0.001	0.316
T × A	(3, 93)	2.789	0.045	0.083
Number of within-object fixations				
Stimulus Type (T)	(1, 31)	32.853	< 0.001	0.515
Angular Disparity (A)	(3, 93)	3.225	0.026	0.094
T × A	(3, 93)	11.929	< 0.001	0.278
Number of saccades				
Stimulus Type (T)	(1, 31)	20.299	< 0.001	0.396
Angular Disparity (A)	(3, 93)	11.343	< 0.001	0.268
T × A	(3, 93)	6.760	< 0.001	0.179
Strategy ratio				
Stimulus Type (T)	(1, 31)	17.008	< 0.001	0.354
Angular Disparity (A)	(3, 93)	9.987	< 0.001	0.244
T × A	(3, 93)	23.035	< 0.001	0.426
